# Exploiting Tissue Dielectric Properties to Shape Microwave Thermal Ablation Zones

**DOI:** 10.3390/s20143960

**Published:** 2020-07-16

**Authors:** Anna Bottiglieri, Giuseppe Ruvio, Martin O’Halloran, Laura Farina

**Affiliations:** 1 Electrical and Electronic Engineering, National University of Ireland Galway, H91 TK33 Galway, Ireland; 2 Translational Medical Device Lab, National University of Ireland Galway, H91 TK33 Galway, Ireland; giuseppe.ruvio@nuigalway.ie (G.R.); laura.farina@nuigalway.ie (L.F.); 3 Endowave Ltd., National University of Ireland Galway, H91 TK33 Galway, Ireland; 4 CÚRAM, SFI Research Centre for Medical Devices, H91 TK33 Galway, Ireland

**Keywords:** microwave ablation, directional heating pattern, biological tissue interface, electromagnetic therapeutics, targeted ablation therapy

## Abstract

The dielectric characterization of tissue targets of microwave thermal ablation (MTA) have improved the efficacy and pre-procedural planning of treatment. In some clinical scenarios, the tissue target lies at the interface with an external layer of fat. The aim of this work is to investigate the influence of the dielectric contrast between fat and target tissue on the shape and size of the ablation zone. A 2.45 GHz monopole antenna is placed parallel to an interface modelled by fat and a tissue characterized by higher dielectric properties and powered at 30 and 60 W for 60 s. The performances of MTA are numerically investigated considering different interface scenarios (i.e., different widths of fat layer, shifts in the antenna alignment) and a homogeneous reference scenario. Experiments (N = 10) are conducted on ex vivo porcine tissue to validate the numerical results. Asymmetric heating patterns are obtained in the interface scenario, the ablation zone in the target tissue is two-fold to ten-fold the size of the zone in the adipose tissue, and up to four times larger than the homogenous scenario. The adipose tissue reflects the electromagnetic energy into the adjacent tissue target, reducing the heating in the opposite direction.

## 1. Introduction

Microwave thermal ablation (MTA) is an alternative therapeutic technique for the treatment of tumors in patients who are not surgical candidates [1,2,3]. In MTA, diseased tissues are destroyed by inducing a high and focused temperature increase (above 55 °C) [4,5]. Localized heating is achieved through tissue absorption of the electromagnetic (EM) energy, at microwave (MW) frequencies [6]. In MTA, the EM field is radiated by an antenna typically powered at 915 MHz or 2.45 GHz [1,7]. The treatment protocol is based on defining the power and time settings of the EM field radiated by the antenna to obtain the desired thermal lesion (i.e., ablation zone) [8]. The clinical objective is to achieve ablative temperatures (at least 55 °C) in the target tissue, while sparing the surrounding healthy tissues [1,2,3,4,5]. Promising results of MTA have been reported for the treatment of hepatocellular carcinomas [9,10], hepatic metastases [11], renal tumors [12,13], lung tumors [14] and bony lesions [15].

Recent studies have presented MTA applicators capable of creating directional patterns to achieve energy deposition limited to preferred directions [16,17,18]. In particular, reflector-based MTA antennas equipped with a variety of reflector shields have been proposed. In McWilliams et al. [16], a monopole antenna equipped with a hemi-cylindrical reflective shield is described. In Sebek et al. [18], two different ablation devices equipped with spherical and parabolic reflectors are proposed. Promising results were obtained on ex vivo bovine liver in terms of localized directional energy deposition. Such MTA applicators have been proposed to meet specific clinical scenarios requiring a side-firing approach. Preservation of healthy tissues around the target region (e.g., diaphragm in peripheral liver lesions [19], spinal cord in metastatic spinal bone tumours [20]) and sparing blood vessels (e.g., renal artery, renal vein, inferior vena cava) are crucial clinical goals to ensure patient safety. However, directional applicators require to be carefully oriented to the target by the surgeon—this procedure is not easy to perform under conventional two-dimensional (2D) imaging guidance.

In this work, we investigate an alternative strategy to focus the radiation energy in a target region by exploiting the typical presence of fat around the target to direct the EM energy. This side-firing approach can be adopted in any peripheral anatomical target surrounded by a layer of visceral fat (like subcapsular hepatic lesions, exophytic renal tumors or adreno-cortical adenomas) using any omnidirectional antenna, hence no orientation by the surgeons is required. Any fat interface is clearly visible through imaging techniques and can be easily adopted to align the MTA applicator, and thus the EM beam, under the guidance of imaging systems currently used in MTA (e.g., Computed Tomography and ultrasound). The interaction between the EM field and the targeted tissue is strictly related to the water content in each tissue, and thus to the dielectric properties (i.e., relative permittivity and effective conductivity) of the biological tissue [21].

The relative permittivity is a function of the ability of the tissue to store energy when an external electric field is applied. The effective conductivity indicates the dissipative nature of the tissue, which absorbs the energy from the applied electric field and partially converts it to heat. The higher the values of relative permittivity and effective conductivity of the tissue, the higher the capabilities of the tissue to store, absorb and convert the applied EM energy to heat. The relative permittivity and effective conductivity gradient lying at the interface between fat (ε_r_ = 10.8, σ = 0.3 S/m at 2.45 GHz) and other tissues can be used to direct the EM radiation and the consequent rise of temperature to the desired target [22,23,24]. As an example, liver, kidney and adrenal glands are characterized by dielectric properties values three to eight times higher than fat. Relative permittivity of liver tissue is about four times that of fat tissue; while in kidney and adrenal gland, the multiplicative factor is five and six, respectively. Effective conductivity of fat is instead four times lower than adrenal gland, about six times lower than liver and about eight times lower than kidney. In this study, muscle and fat tissues were adopted to investigate the effect of the dielectric contrast on the shaping of the ablation zone. Compared to the above-mentioned clinical targets, muscle represents a conservative model: relative permittivity and effective conductivity of muscle are four and three times higher than fat, i.e., the dielectric contrast is smaller compared to liver, kidney or adrenal gland.

A comprehensive proof-of-concept study including both numerical and experimental evaluations was conducted to validate the feasibility of exploiting the fat layer to purposely and effectively direct the ablation zone. In the numerical study, the fat and muscle tissues were modelled by two layers represented by the corresponding dielectric properties acquired at room temperature. The results obtained in the two-layer scenario were compared with a homogeneous scenario, to highlight the asymmetry obtained. An interstitial MTA monopole antenna was designed and optimized for the study. The numerical tests were experimentally validated: the designed antenna was built and ex vivo experiments were conducted exploiting the natural interface between fat and muscle using porcine tissue samples.

The remainder of this work is organized as follows: Section 2 describes the numerical and the experimental methodology. In Section 3, the results in terms of antenna performance and temperature distributions are shown. In Section 4, the obtained results are discussed, comparing the numerical and experimental outcomes in terms of the temperature distributions. Finally, in Section 5, the conclusions of this work are presented.

## 2. Materials and Methods

### 2.1. Numerical Study

**Figure 1:**
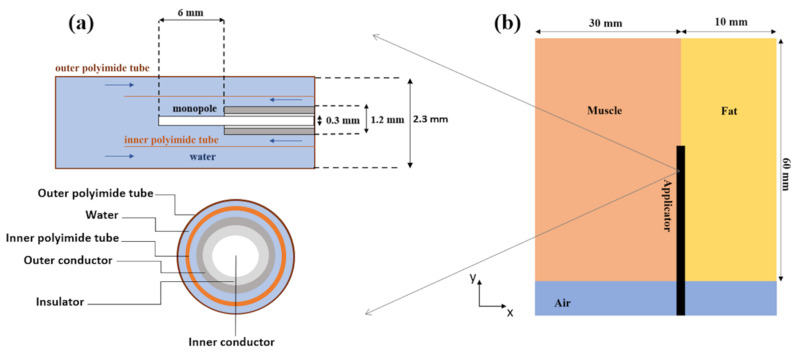
(**a**) Longitudinal section (top) and cross-section (bottom) geometry of the distal portion of the interstitial MW antenna: the 6 mm radiating element and the integrated cooling system are depicted. The inbound and outbound water flows are illustrated (blue arrows). Both the inner tube, that provides the onward water flow, and the outer wall of the catheter are made of polyimide material. (**b**) Scheme of the applicator (MW antenna) placed parallel to the interface between two materials (muscle and fat) characterized by different dielectric and thermal properties.

An 18-gauge monopole antenna was modelled using a 3D (three-dimensional) commercial full-wave electromagnetic software (CST Microwave Studio (MWS) Suite 2018, Darmstadt, Germany). A simulation-based approach involving a parametric sweep across the range of 0.5–3 GHz was employed to optimize the length of the radiating element in order to obtain the minimum reflection coefficient (S11) for the operating frequency of 2.45 GHz. A diagram of the designed applicator is depicted in Figure 1a: two concentric polyimide tubes were used to model an integrated cooling system. The water flow proximal to the antenna radiating element allowed heat dissipation within the lossy cables, limiting heating within and close to the applicator length. Convective boundary conditions along the feeding cable of the antenna were considered to model the cooling system of the antenna: the convection coefficient was set equal to 1000 (W m^−2^ °C^−1^) and the water temperature was equal to 18 °C.

The above-mentioned CST MWS commercial software was used to conduct coupled electromagnetic and thermal simulations. Firstly, a numerical study was conducted in a homogeneous muscle scenario and then followed a more complex interface scenario. The ablation applicator was placed parallel to the interface between two biological tissues (i.e., fat and muscle), as sketched in Figure 1b. In the homogeneous scenario, the applicator was placed at the center of a muscle-mimicking 3D geometry (x = 60 mm, y = 60 mm, z = 60 mm). For the interface scenario, a multilayer 3D geometry was developed including two adjacent cuboids mimicking muscle and fat dielectric tissues, characterized respectively by high and low dielectric properties values. The muscle layer was 30 mm wide, whereas the fat was modelled by a 10 mm wide layer. The influence of the fat layer width was investigated considering two scenarios where the width of the fat layer was increased up to 15 mm and reduced to 5 mm. The influence of the antenna alignment was investigated by shifting the antenna position of 1.5 mm both in muscle and in fat along the *x*-axis, with reference to the initial position shown in Figure 1b.

The software solves the Helmholtz electromagnetic wave equation:

(1) $$\nabla^{2}E-k_{0}^{2}\left(\varepsilon_{r}-\frac{j\sigma }{\omega \varepsilon_{0}}\right)E=0$$

where ***E*** (V m^−1^) is the electric field, $k_{0}$ (m^−1^) is the wave-number in the free-space, $\varepsilon_{r}$ is the relative permittivity, σ (S m^−1^) is the effective conductivity, ω (rad s^−1^) is the angular frequency and $\varepsilon_{0}$ (F m^−1^) is the permittivity of the free-space.

From the electromagnetic simulation, the specific absorption rate (SAR) distribution in the tissue is calculated using (2):

(2) $$SAR=\frac{\sigma \;{\left|E\right|}^{2}}{2\rho }$$

where σ (S m^−1^) is the effective conductivity, ***E*** (V m^−1^) is the intensity of the electric field and ρ (kg m^−3^) is the density.

Then, Pennes’ bioheat equation [25] is employed to describe the heat transfer inside the model and to predict the resulting temperature distribution in the tissue

(3) $$\rho c\frac{\partial T}{\partial t}=\nabla \cdot \left(k\nabla T\right)+\rho Q+\rho SAR-m_{b}c_{b}\left(T-T_{b}\right)\;$$

where ρ (kg m^−3^) is the density of the biological tissue, c (J kg^−1^ K^−1^) is the specific heat capacity of the tissue, *T* (K) is the temperature, *t* (s) is the time, k (W K^−1^ m^−1^) is the thermal conductivity of the biological tissue, Q (W kg^−1^) is the metabolic heat generation rate, SAR (W kg^−1^) is the rate of absorption of the electromagnetic power by the biological tissues, $m_{b}$ (kg m^−3^ s) is the blood mass perfusion rate, $c_{b}$ is the specific heat capacity of the blood and $T_{b}$ is the temperature of the blood. The ex vivo condition was obtained in Equation (3) discarding the contribution of the metabolic heat (ρQ) and the blood perfusion ($m_{b}c_{b}\left(T-T_{b}\right)$).

Scattering boundary conditions were applied to the outer surface of the modelled region to minimize the reflected EM waves. The models were meshed in order to have denser mesh in the regions with higher permittivity. First, a minimum meshing size was considered (i.e., ten cells per wavelength), then the number of cells per wavelength was increased until a discrepancy in the S11 values less than 0.1% was obtained between two consecutive simulations. Finally, the tissue models were discretized using tetrahedrons with a size between 0.2 and 3.3 mm.

The thermal and dielectric properties used for the tissue models are detailed in Table 1 at 2.45 GHz. The dielectric properties were obtained experimentally and loaded into the material library of the CST MWS software: measurements were conducted on ex vivo porcine tissue using a Keysight slim form probe (N1501A) connected to a Keysight E5063A network analyzer (operating frequency range: 100 kHz–8.5 GHz) as described in La Gioia et al. [26]. The values related to heat capacity, thermal conductivity and density were obtained from the literature [22] and manually loaded into the material settings of the CST MWS software.

For the tissue models, the starting temperature was set to 25 °C. The effect of two power levels commonly used [18,23] were compared over 60 s: 30 W and 60 W.

The values obtained from each numerical simulation were exported and analyzed in MATLAB (R2017a, The MathWorks, Inc., Natick, MA, USA). The error introduced for the representation of the ablation profiles is around 0.1%.

### 2.2. Experimental Study

The designed monopole antenna was then fabricated exposing 6 mm from the inner conductor of a UT-047 semirigid coaxial cable (Micro-Coax Inc., Pottstown, PA, USA). A SMA connector was placed at 165 mm from the antenna feed-point [27].

The impedance matching of the fabricated antenna was evaluated in terms of reflection coefficient (S11) by connecting the antenna to an antenna analyzer (Rohde & Schwarz^®^ ZVH8 100 kHz–8 GHz, Munich, Germany) through a coaxial cable. A full one-port calibration procedure was performed, considering three different calibration standards: open circuit, short circuit and 50 Ω load. Five measurements were conducted loading the test device (ablation antenna) with water at room temperature. The S11 values were recorded at 201 linearly spaced frequency points over 0.5–3 GHz.

After this preliminary step, experiments were conducted on ex vivo porcine tissue to validate the results of the numerical study. The temperature of the material under test (porcine tissue) was measured using an infrared thermometer (Fluke 62 Max IR Thermometer, −30–500 °C temperature range, accuracy of 1.5 °C of reading at temperature ≥ 0 °C, Fluke Corporation, Everett, WA, USA). The applicator was placed along the interface between fat and muscle layers and covered by another layer of tissue characterized by the same pattern. Two power and time settings were experimentally considered: 30 W for 60 s and 60 W for 60 s. The ablations were conducted with 32 W and 64 W as output powers at the microwave generator (Sairem, SAS, Décines-Charpieu, France) at 2.45 GHz.

The selected powers correspond to 30 W and 60 W at the antenna feed point, considering 1.9 dB/m losses calculated along the coaxial cable with reference to the cable datasheet [28]. Five ablations were conducted for each setting, yielding a total of ten experiments. A peristaltic dispensing pump (DP2000, Thermo-Fisher Scientific Inc., Waltham, MA, USA) was connected to the inflow channel of the ablation applicator, operating at 50 mL/min. For each experiment, the temperature was monitored using three fiber optic sensors (Neoptix Inc., Québec, QC, Canada) placed at three different distances from the antenna axis (reference axis) in correspondence with the antenna feed. Two fiber optic sensors were placed at a radial distance of 4 mm from the antenna feed in the muscle tissue and in the fat tissue, in order to consider the increase of the temperature caused by the direct heating in proximity to the antenna. One fiber optic sensor was placed at 7 mm from the antenna feed in the muscle, in order to account for the heat transfer by diffusion phenomena through the tissue (i.e., muscle) at a higher distance from the antenna feed. At *t* = 0 s, the power supply from the MW generator was switched on, and the temperature was monitored over 60 s.

## 3. Results

The MTA antenna performance was evaluated in terms of magnitude of the reflection coefficient (S11) and resonance bandwidth within the 0.5–3 GHz frequency range, numerically as well as experimentally, in water. The reflection coefficient of the antenna is equal to −12.6 dB and −22.4 dB at 2.45 GHz for experimental and numerical results, respectively. A comparable −10 dB antenna bandwidth is obtained between 1.9 and 2.5 GHz experimentally, and between 1.8 and 2.9 GHz numerically.

**Figure 2:**
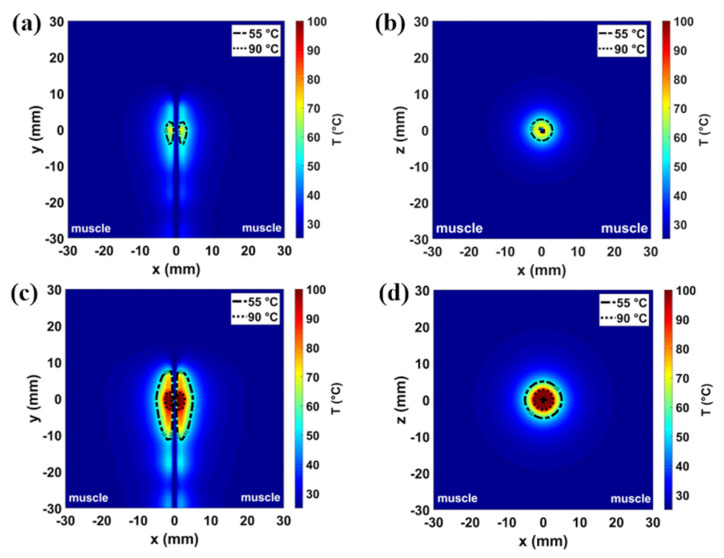
Simulated temperature patterns in the homogeneous muscle scenario shown both in a coronal plane (first column) and transverse plane (second column) with reference to the antenna feed. Two different power-time settings are reported: 30 W–60 s (**a**,**b**) and 60 W–60 s (**c**,**d**). The dash-and-dot lines represent 55 °C boundary and the dot lines represent 90 °C boundary.

**Figure 3:**
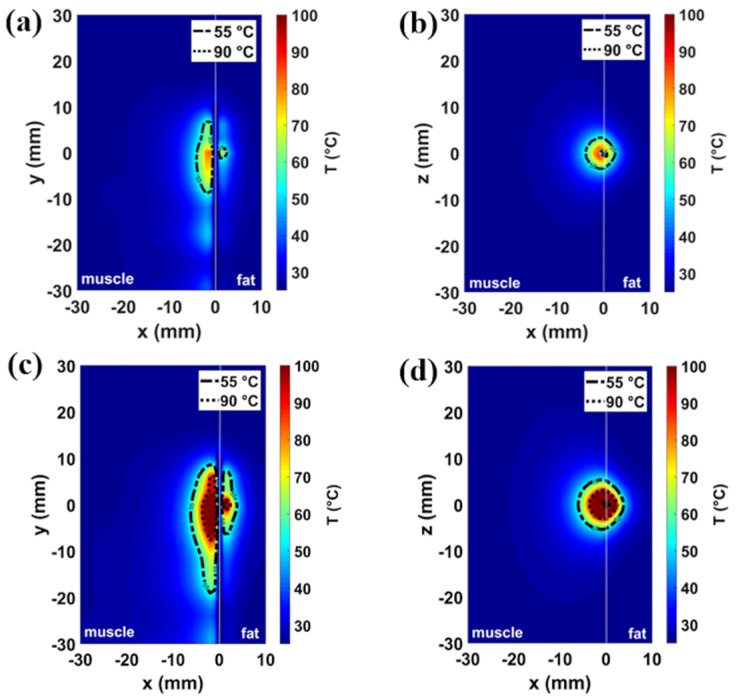
Simulated temperature patterns in muscle-fat interface scenario shown both in a coronal plane (first column) and transverse plane (second column) with reference to the antenna feed. Two different power-time settings are reported: 30 W–60 s (**a**,**b**) and 60 W–60 s (**c**,**d**). The dash-and-dot lines represent 55 °C boundary and the dot lines represent 90 °C boundary.

**Figure 4:**
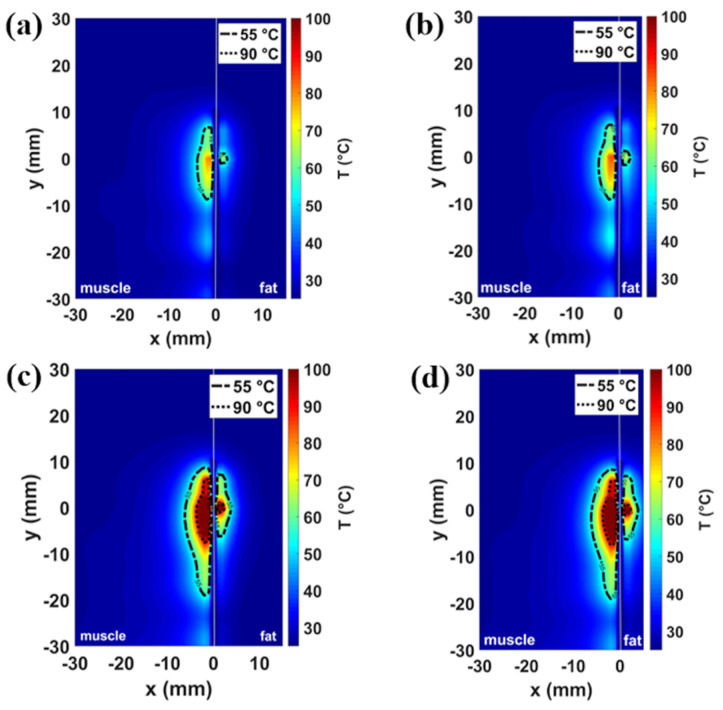
Simulated temperature patterns in muscle-fat interface scenario shown in a coronal plane with reference to the antenna feed for two different fat layer widths: 15 mm (**a**,**c**) and 5 mm (**b**,**d**). Two different power-time settings are reported: 30 W, 60 s (**a**,**b)**, and 60 W, 60 s (**c**,**d**).

**Figure 5:**
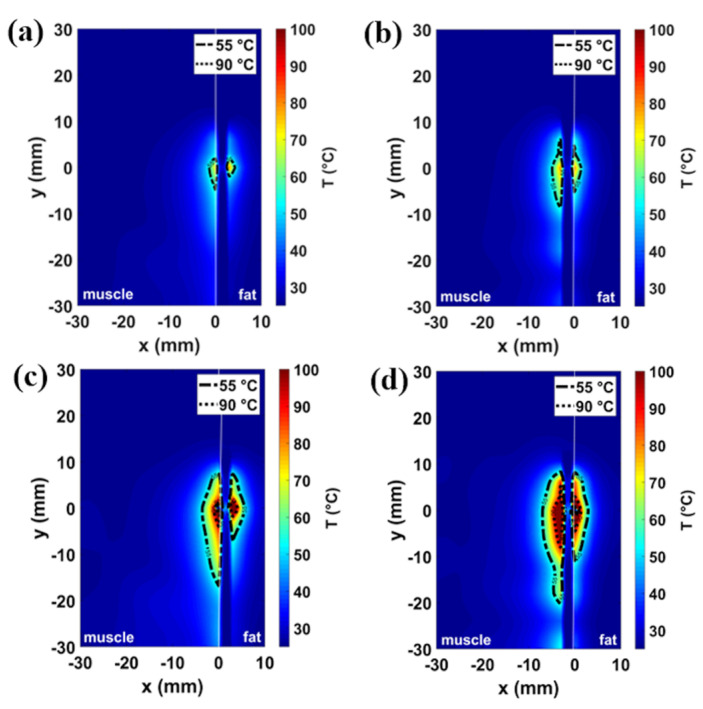
Simulated temperature patterns in muscle-fat interface scenario shown in a coronal plane with reference to the antenna feed for 1.5 mm displacement of the antenna along x-axis in fat (**a**,**c**) and in muscle (**b**,**d**). Two different power-time settings are reported: 30 W, 60 s (**a**,**b**) and 60 W, 60 s (**c**,**d**).

The numerical results related to different tissue models and antenna placements are illustrated in Figure 2, Figure 3, Figure 4 and Figure 5. The thermal distributions achieved after 60 s in each tissues model are mapped for both the power settings considered, i.e., 30 W and 60 W. Two temperature profiles are highlighted: a contour line at 55 °C marks the area where instantaneous coagulation occurs (i.e., ablation zone), while the 90 °C contour line indicates the area where water vaporization in the tissue may occur [29,30,31].

Figure 2 and Figure 3 show the temperature profiles obtained in the homogeneous muscle model and in the muscle-fat interface model (10 mm fat layer), respectively. In particular, the temperature profiles related to the 30 W for 60 s setting are depicted in Figure 2a,b and Figure 3a,b, while the temperature profiles related to the 60 W for 60 s setting are depicted in Figure 2c,d and Figure 3c, d. The asymmetry introduced by the inhomogeneity of the tissue (Figure 3) with respect to the homogeneous muscle scenario (Figure 2) can be observed. For the low power setting (30 W for 60 s), the increase of temperature in the tissue close to the antenna is below 90 °C both in the homogenous scenario of Figure 2a,b and in the interface scenario of Figure 3a,b. The area enclosed in the 55 °C contour is 10 mm^2^ each side (20 mm^2^ overall) in the case of homogeneous scenario; whereas in the case of the interface scenario, the extent of the area exceeding 55 °C is 3 mm^2^ in fat and 41 mm^2^ in muscle. Increasing the input power (60 W for 60 s), the MW heating affects larger areas both in muscle homogeneous and in muscle-fat interface scenario, as shown in Figure 2c,d and Figure 3c,d, respectively. In the case of the 60 W setting, temperatures higher than 90 °C are found in proximity to the antenna feed both in the homogeneous muscle and muscle-fat interface scenarios. However, temperature values exceeding 90 °C result in a smaller area in fat (<3 mm^2^) compared to the muscle (<30 mm^2^) in the case of the muscle-fat interface model. The area exceeding 55 °C is around 65 mm^2^ each side (130 mm^2^ overall) in the homogeneous muscle model; whereas in the interface scenario, they are approximately 31 and 117 mm^2^, in fat and in muscle, respectively. Comparing the two settings, we can observe that the ablation area in muscle in the interface scenario at 30 W is about ten times greater than the zone area in fat and four times the zone area in muscle (one side) in the homogeneous scenario; while in the interface case at 60 W, the ablation area in muscle is about four times the zone area in fat and two times the zone area in muscle in the homogeneous case.

Figure 4 shows the impact of the fat layer width on the profiles of the ablation zone: the temperature distributions obtained in the presence of 5 and 15 mm-wide fat layers are illustrated for the two different settings proposed (30 W in Figure 4a,b and 60 W in Figure 4c,d). No significant variations in the extent of the ablation zone compared to the scenario of the 10 mm fat layer are observed. The areas exceeding 55 °C are 39 mm^2^ for 30 W, 60 s, and 112 mm^2^ for 60 W, 60 s, in muscle, 3 mm^2^ for 30 W, 60 s, and 30 mm^2^ for 60 W, 60 s, in fat, for the 15 mm case, and 43 mm^2^ for 30 W, 60 s, and 114 mm^2^ for 60 W, 60 s, in muscle, and 4 mm^2^ for 30 W, 60 s, and 31 mm^2^ for 60 W, 60 s, in fat, for the 5 mm case. The ablation areas calculated both at 30 W and 60 W power settings for the homogeneous scenario and for each muscle-fat interface scenario are reported in Table 2.

Figure 5 shows the results obtained for 30 W, 60 s (Figure 5a,b) and 60 W, 60 s (Figure 5c,d) settings accounting for the influence of an antenna misalignment of 1.5 mm in fat (Figure 5a,c) or muscle (Figure 5b,d). In the case of 1.5 mm displacement of the antenna in fat, the longitudinal axis of the antenna is positioned inside the fat layer, thus the antenna is immersed in fat. In the case of 1.5 shift of the antenna in muscle, the longitudinal axis of the antenna is positioned inside the muscle region, thus the antenna is immersed in muscle.

In the case of 1.5 mm misalignment in fat, the sizes of the ablation zones are 7 mm^2^ in muscle and 4 mm^2^ in fat for the 30 W power setting and 67 mm^2^ in muscle and 28 mm^2^ in fat for the 60 W setting. For the antenna misalignment in muscle, the areas exceeding 55 °C are 20 mm^2^ in muscle and 10 mm^2^ in fat for 30 W, and 89 mm^2^ in muscle and 47 mm^2^ in fat for 60 W. Compared to the scenario of perfect alignment of the antenna along the interface between fat and muscle (Figure 3), a smaller asymmetry can be observed: the ablation areas in muscle are only two times the ablation areas in fat. For both the misalignment scenarios, a smaller impact of the fat-muscle interface on the reflection of the electromagnetic energy in the target tissue is observed.

**Figure 6:**
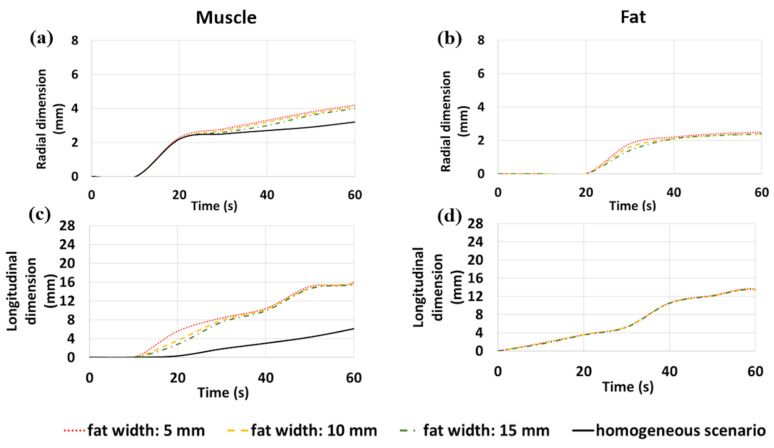
Variations of the radial (**a**,**b**) and longitudinal (**c**,**d**) dimensions of the ablation zone over time at 30 W in muscle (**a**,**c**) and in fat (**b**,**d**). Interface scenarios (red line: 5 mm wide fat case; yellow line: 10 mm wide fat case; green line: 15 mm wide fat case) and homogenous scenario (black line) are compared.

**Figure 7:**
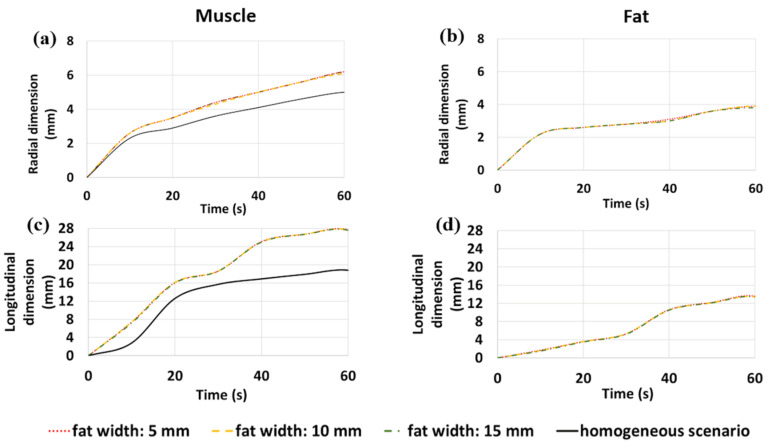
Variations of the radial (**a**,**b**) and longitudinal (**c**,**d**) dimensions of the ablation zone over time at 60 W in muscle (**a**,**c**) and in fat (**b**,**d**). Interface scenarios (red line: 5 mm wide fat case; yellow line: 10 mm wide fat case; green line: 15 mm wide fat case) and homogenous scenario (black line) are compared.

The numerical results presented above (Figure 2, Figure 3 and Figure 4) are compared in Figure 6 and Figure 7 in terms of variations of the radial and longitudinal dimensions of the ablation zone (i.e., the area where the temperatures is above 55 °C) over the time of the ablation procedure (up to 60 s, at a 10 s time step). The dimensions are reported for both fat and muscle of the interface scenario, for each fat width considered (5, 10, 15 mm) and for the homogenous scenario. The increases in the radial and longitudinal dimensions of the ablation zone over time in muscle for each one of the different fat layer widths investigated can be observed in comparison with the homogeneous scenario. First, it can be noticed that the growth rate of the ablation zone is independent of the fat width in both tissues.

In the case of the 30 W setting (Figure 6), the ablation zone starts to form only after 10 s in muscle and 20 s in fat. The radial dimension of the ablation zone in muscle at the interface with fat starts to increase after 20 s with respect to the homogeneous scenario, while such difference becomes sizeable after about 40 s. In fat, the radial dimension of the ablation zone reaches the steady state after 40 s. Longitudinally, the growth of the ablation zone in fat is limited during the whole procedure, while a substantial increase of the ablation zone in muscle with respect to the homogeneous case is observed. In particular, the longitudinal dimension in muscle grows about 0.3 mm/s, steadily from 10 to 40 s. At 40 s, a change in the slope of the curve occurs and an increase of about 5 mm is observed at 50 s (about 0.5 mm/s), before reaching a plateau.

In the case of the 60 W setting (Figure 7), higher values of the radial dimension of the ablation zone in muscle compared to the homogeneous scenario are visible after 10 s, and this difference remains constant over time. In fat, the radial dimension of the ablation zone reaches a plateau after 10 s and slowly starts increasing again after 40 s. In contrast to the 30 W case, the growth of the ablation zone along the longitudinal dimension is noticeable in fat at 60 W. Longitudinally, the ablation zone in fat is limited for the first 30 s; then, at 30 s, a change in the slope of the curves both in fat and muscle leads to a substantial increase in the ablation zone length over the following 30 s (up to the end of the procedure). A similar change of slope can be observed in muscle at the same time (at 30 s).

**Figure 8:**
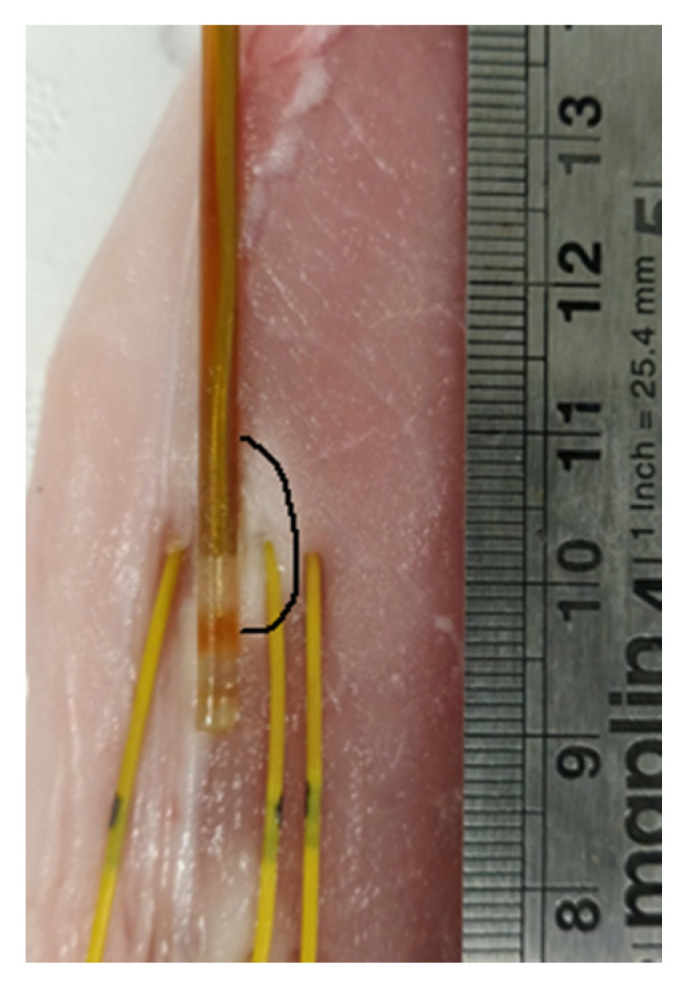
Section of a MTA obtained in an ex vivo porcine sample: the applicator (MW antenna) and fiber optic sensors’ placement in the tissue is shown. The antenna is placed at the visible interface between the muscle and the fat, and the ablation zone obtained in the muscle is marked. The ablation zone is not visible in fat.

**Figure 9:**
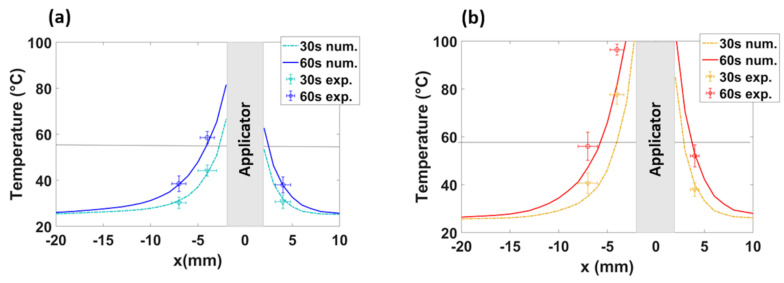
Numerical (num.) and experimental (exp.) values of temperature over the radial distance from the MW antenna axis (applicator) both in muscle and fat at 30 s and 60 s for 30 W (**a**) and 60 W (**b**). The horizontal line highlights the temperature threshold at 55 °C.

Finally, the numerical results have been experimentally validated. Experimental MTAs (N = 10, five at 30 W for 60 s and five at 60 W for 60 s) were obtained, exploiting the natural muscle-fat interface of ex vivo porcine tissue. The MW antenna was placed adjacent to the interface between muscle and fat, and the increase of temperature at different radial distances from the antenna axis was monitored with fiber optic sensors placed in fat at 4 ± 1 mm from the antenna axis and in muscle at 4 ± 1 mm and at 7 ± 1 mm from the antenna axis (as shown in Figure 8). The thermal profiles observed numerically at 30 and 60 s are compared with the experimental values obtained from the fiber optic sensors at 30 and 60 s during the MTA procedure in Figure 9 for 30 W (Figure 9a) and 60 W (Figure 9b). The temperature increase as a function of the radial distances from the antenna feed is reported. The experimental data well match the numerical trends and the impact of the differences in electrical and thermal characteristics between two tissues is visible.

## 4. Discussion

In this work, the influence of the fat layer tissue on the size and shape of microwave ablation zones as well as on the temperature increase is characterized. The aim of the study was to investigate the role of the fat layer, characterized by dielectric and thermal properties lower than the target tissue, as a biological “shield” of the heating pattern, i.e., the ability of fat to reflect the electromagnetic power into the tissue target while reducing the heating in the opposite direction. This approach could be exploited for the minimally invasive treatment of peripheral lesions (e.g., in liver or kidney) and adenomas affecting adrenal glands, where an interface between fat and the target tissue is available and accessible with the antenna parallel to the interface [32]. Such an interface (fat-target tissue) can be easily detected by imaging techniques, used to align the antenna and perform a side-firing ablation without the need for the clinicians to carefully manage the antenna orientation (as with directional antennas). A similar concept has been adopted in Kastler et al. [20], for the MTA treatment of spinal metastatic tumors, where the low dielectric properties of the adjacent vertebral bone facilitated the local control of the ablation zone in the surrounding neural structures.

An investigative numerical study was proposed in this work exploiting an exemplificative and dielectrically conservative interface model (muscle-fat) and a monopole antenna. The numerical analysis was validated in an ex vivo porcine model with a monopole antenna prototyped in-house. First, the ability of the antenna to effectively transfer the power to the tissues was ensured. A reflection coefficient below −10 dB over a sufficiently large frequency interval centered at the operating frequency of 2.45 GHz was secured, in order to guarantee satisfying operational performance independently of the variations in dielectric properties of the tissue.

The optimized antenna was adopted to conduct the numerical study both in the muscle-fat interface model and in the homogeneous model. Comparing the results obtained in the homogenous model (Figure 2) with the results obtained in the muscle-fat interface model (Figure 3), asymmetry in the heating distributions are observed at the interface between the adipose layer and the muscle layer. The asymmetric heating patterns observed for each power and time setting (Figure 3) are the result of the different interaction mechanisms between fat and muscle with the EM field. Because of the lower values in relative permittivity and effective conductivity, ablation zones (i.e., the area where a temperature at least equal to 55 °C is achieved) in the fat layer are observed to be constantly smaller and more spherical compared to the muscle layer, independently from the thickness of the fat layer (Figure 4). Different ratios of asymmetry were observed for the two power settings investigated: the ablation zone in fat was at least ten times smaller than in muscle for the lower power tested (30 W), and about four times for the higher power (60 W).

The high contrast in terms of relative permittivity and effective conductivity between fat and muscle at the interface induces higher levels of energy, and consequently of heating, in the muscle layer in proximity to the applicator, when compared to the homogeneous scenario. Accordingly, it is conceivable that the fat layer not only shields the electromagnetic radiation, but also reflects the electromagnetic power into the adjacent tissue characterized by higher values of relative permittivity and effective conductivity. The electromagnetic field reflected in muscle, at the interface with fat, is redistributed along the antenna axis, inducing a noticeable, yet not desirable, elongation of the ablation zone in muscle. In the used applicator, back-heating along the antenna axis is limited only by the active cooling that balances the temperature increase. Thus, such increase in the longitudinal direction would be expected, but could be easily minimized adopting a different antenna design [33,34].

Moreover, different rates of coagulation (depicted in Figure 6 and Figure 7 by the edge of the ablation zone, i.e., by the 55 °C threshold) were observed for the two different levels of input power (30 and 60 W) in muscle in the interface scenario, when compared with the homogeneous scenario. In particular, a sequence of saturations (plateau) of the monotonic increase of the length of the ablated zone (longitudinal dimension of Figure 6 and Figure 7) have been observed at different times for the two different power settings proposed. Both at 30 and 60 W, the change in the slope of the curves related to the longitudinal dimension of the ablation zone occurs when the radial dimension of the ablation zone in muscle in the interface scenario becomes higher than the equivalent dimension obtained in the homogeneous scenario. Therefore, at 40 s in the case of 30 W and at 30 s in the case of 60 W, the ablation length in muscle increases faster than the corresponding one in the homogenous scenario, inducing the elongation of the ablation zones. A similar behavior has been observed for MTA in thin tissue samples [35]. These differences suggest that the success of this approach is highly dependent on the power and time settings of the procedure: lower power and shorter ablation time enable more controlled ablation zones and promising results. Pulsed protocols could be optimal for this approach in order to allow the fat tissue to recover from the heat stress and retrieve its shielding capabilities. The higher dielectric properties (i.e., relative permittivity and effective conductivity) of muscle enable higher deposition of the electromagnetic power and temperature increases compared to fat; while, the different thermal conductivities between fat and muscle determine the steepness of the temperature increase. Thus, the reduced heating in the adipose tissue is also due to the lower values of thermal conductivity and thermal diffusivity (i.e., ratio between thermal conductivity and volumetric heat capacity). In particular, the lower diffusivity of the fat tissue is responsible for the slower propagation of the heat beyond the area of active heating due to the direct deposition of the electromagnetic power.

Antenna misalignment was investigated to account for a scenario of poor spatial resolution provided by the imaging technique currently used [36]. Asymmetric heating patterns were numerically obtained also in the case of a not perfect alignment of the antenna at the interface between fat and muscle, even if the difference between the two adjacent ablation zones was visibly reduced (Figure 5). A two-fold asymmetry was observed independently from the power applied (instead of the ten- and four-fold observed for the perfect alignment at 30 and 60 W, respectively). The precise orientation of the antenna is not strictly required to exploit the shielding effect induced by the fat presence, but it should be considered to properly predict the resulting dimension of the ablated zone.

Finally, the numerical results were successfully validated experimentally. The temperature profiles monitored in both tissues (Figure 9) are in good agreement with the patterns predicted by the thermal simulations. A minimal discrepancy between the numerical and experimental data can be observed in muscle only for the 60 W MTA setting. This mismatch could be likely related to the transient deformations of the tissue in proximity to the antenna. Such tissue deformation (i.e., shrinkage) was not considered in the numerical model [37,38]. Moreover, the thermal lesions obtained from the numerical simulations were calculated considering the minimum temperature equal to 55 °C, i.e., an approximated value. Such value was chosen in this study considering that the minimum temperature to obtain the coagulation effect of the target tissue is reasonably within 50–60 °C [1]. However, this value can slightly vary with the tissue types and due to different initial conditions (e.g., initial temperature) of the tissue.

## 5. Conclusions

This work analyzes the impact of the fat layer on the coagulated zone achievable through microwave thermal ablation procedures. Results show that, when a layer of fat tissue lies at the interface with a tissue characterized by higher dielectric properties (muscle tissue was used in this study), the area of the ablation zone increases with respect to the homogeneous scenario (only muscle). Such an increase is caused by dielectric contrast between the two adjacent tissues and the consequent reflection of the electromagnetic field into the tissue characterized by higher dielectric properties (i.e., the target) compared to the shield tissue (i.e., fat). The findings of this study highlight the potential advantage to exploit the natural fat layer at the interface with potential targets, such as liver, kidneys and adrenal glands to focus the most part of the electromagnetic field into the target tissue.

## Figures and Tables

**Table 1:** Tissue dielectric and thermal properties employed in the numerical simulations.

Parameter	Fat	Muscle
Relative Permittivity, ε_r_	8.7	41.0
Effective Conductivity, σ_eff_ (S/m)	0.1	0.7
Volumetric Heat Capacity, *ρ*c (kJm^−3^K^−1^)	2139	3729
Thermal Conductivity, k (Wm^−1^K^−1^)	0.2	0.5
Frequency (GHz)	2.45	

**Table 2:** Areas of ablation zones calculated at 30 W (first line) and at 60 W (second line) in muscle in the case of the homogeneous scenario and both in muscle and in fat in the interface scenario considering 15, 10 and 5 mm fat layer width.

Settings		Area Enclosed in 55 °C Contour						
		Homogeneous	Interface Fat Width: 15 mm		Interface Fat Width: 10 mm		Interface Fat Width: 5 mm	
**P** **(W)**	**t** **(s)**	Muscle *	**Fat**	**Muscle**	**Fat**	**Muscle**	**Fat**	**Muscle**
30	60	10 mm^2^	3 mm^2^	39 mm^2^	3 mm^2^	41 mm^2^	4 mm^2^	43 mm^2^
60	60	65 mm^2^	30 mm^2^	112 mm^2^	31 mm^2^	117 mm^2^	31 mm^2^	114 mm^2^

* in the case of homogeneous scenario, the values reported refer to one side of the muscle.
